# Increased plasma neurofilament light chain concentration correlates with severity of *post-mortem* neurofibrillary tangle pathology and neurodegeneration

**DOI:** 10.1186/s40478-018-0649-3

**Published:** 2019-01-09

**Authors:** Nicholas J. Ashton, Antoine Leuzy, Yau Mun Lim, Claire Troakes, Tibor Hortobágyi, Kina Höglund, Dag Aarsland, Simon Lovestone, Michael Schöll, Kaj Blennow, Henrik Zetterberg, Abdul Hye

**Affiliations:** 1 Department of Psychiatry and Neurochemistry, Institute of Neuroscience & Physiology, the Sahlgrenska Academy at the University of Gothenburg, Mölndal, Sweden; 2 Wallenberg Centre for Molecular and Translational Medicine, University of Gothenburg, Gothenburg, Sweden; 3 King’s College London, Institute of Psychiatry, Psychology & Neuroscience, Maurice Wohl Clinical Neuroscience Institute, London, UK; 4 NIHR Biomedical Research Centre for Mental Health & Biomedical Research Unit for Dementia at South London & Maudsley NHS Foundation, London, UK; 5 MTA-DE Cerebrovascular and Neurodegenerative Research Group, Department of Neurology, University of Debrecen, Debrecen, Hungary; 6 Centre for Age-Related Medicine, Stavanger University Hospital, Stavanger, Norway; 7 Department of Psychiatry, University of Oxford. Warneford Hospital, Oxford, UK; 8 Clinical Memory Research Unit, Department of Clinical Sciences, Malmö, Lund University, Lund, Sweden; 9 Department of Neurodegenerative Disease, UCL Institute of Neurology, London, UK; 10 Clinical Neurochemistry Laboratory, Sahlgrenska University Hospital, Mölndal, Sweden; 11 Department of Molecular Neuroscience, UCL Institute of Neurology, Queen Square, London UK; 12 UK Dementia Research Institute at UCL|, London, UK

**Keywords:** Neurofilament light chain, Blood biomarkers, Braak, Tau, Post-mortem

## Abstract

Alzheimer’s disease (AD) is pathologically characterized by the accumulation of amyloid-β (Aβ) plaques, neurofibrillary tangles and widespread neuronal loss in the brain. In recent years, blood biomarkers have emerged as a realistic prospect to highlight accumulating pathology for secondary prevention trials. Neurofilament light chain (NfL), a marker of axonal degeneration, is robustly elevated in the blood of many neurological and neurodegenerative conditions, including AD. A strong relationship with cerebrospinal fluid (CSF) NfL suggests that these biomarker modalities reflect the same pathological process. Yet, the connection between blood NfL and brain tissue pathology has not been directly compared. In this study, longitudinal plasma NfL from cognitively healthy controls (*n* = 12) and AD participants (*n* = 57) were quantified by the Simoa platform. On reaching *post-mortem*, neuropathological assessment was performed on all participants, with additional frozen and paraffin-embedded tissue acquired from 26 participants for further biochemical (Aβ_1–42_, Aβ_1–40_, tau) and histological (NfL) evaluation. Plasma NfL concentrations were significantly increased in AD and correlated with cognitive decline, independent of age. Retrospective stratification based on Braak staging revealed that baseline plasma NfL concentrations were associated with higher neurofibrillary tangle pathology at *post-mortem*. Longitudinal increases in plasma NfL were observed in all Braak groupings; a significant negative association, however, was found between plasma NfL at time point 1 and both its rate of change and annual percentage increase. Immunohistochemical evaluation of NfL in the medial temporal gyrus (MTG) demonstrated an inverse relationship between Braak stages and NfL staining. Importantly, a significant negative correlation was found between the plasma NfL measurement closest to death and the level of NfL staining in the MTG at *post-mortem*. For the first time, we demonstrate that plasma NfL associates with the severity of neurofibrillary tangle pathology and neurodegeneration in the *post-mortem* brain.

**Electronic supplementary material:**

The online version of this article (10.1186/s40478-018-0649-3) contains supplementary material, which is available to authorized users.

## Introduction

In recent years, there has been an increasing emphasis on developing a blood biomarker (plasma or serum) to predict the clinical onset of Alzheimer’s disease (AD) or to identify the underlying pathophysiology at its earliest stage. A blood-based measure has substantial practical and economic advantages over the most well-established AD biomarkers. Structural imaging (MRI) and positron emission tomography (PET) techniques using ^18^F-fluorodeoxyglucose, amyloid tracers, and, more recently, tau ligands are costly, and access is limited to specialised centres. On the other hand, cerebrospinal fluid (CSF) sampling is becoming routine in neurology clinics and the cost for the core AD CSF immunological assays (Aβ_1–42_, phosphorylated tau [P-tau] and total tau [T-tau]) are much lower per patient than for PET scans. Yet, there remains a level of perceived invasiveness or complexity attached to a lumbar puncture in many countries. Therefore, a blood-based marker would complement CSF and molecular imaging biomarkers as a simplified initial triage step in a multi-stage assessment for early diagnosis, secondary prevention trial participant selection or monitoring of response to intervention over time (for review see, (Lewczuk et al. 2017)).

It has proven challenging to establish a robust blood biomarker for AD. Candidate proteins are influenced by peripheral expression from extra-cerebral tissues and the measurements are confounded by matrix effects from plasma proteins (Zetterberg 2015). More recently, ultra-sensitive immunological assays (Single Molecule Array) and mass spectrometric studies have overcome these complications and recently reported plasma amyloid-beta peptide ratios (Aβ_1–42_/Aβ_1–40_ and APP_699–711_/Aβ_1–42_) that can identify cerebral amyloidosis with high accuracy (Janelidze et al. 2016; Nakamura et al. 2018). Furthermore, using the Simoa platform, plasma T-tau has been shown to be consistently elevated in AD (Mattsson et al. 2016) and to correlate with cognitive decline (Mielke et al. 2017). However, there are large overlaps in concentrations between clinical groups, with a poor association with CSF T-tau (Mattsson et al. 2016; Zetterberg et al. 2013). One promising avenue for AD is the neuroaxonal injury marker neurofilament light (NfL). Patients with AD have increased NfL concentrations in the CSF (Sjogren et al. 2000) and this is also reflected in the blood despite being more than 50-fold lower in concentration (Gisslen et al. 2016; Lewczuk et al. 2018). Increases in blood NfL have also been described in frontotemporal dementia (Rohrer et al. 2016), Huntington’s disease (Byrne et al. 2017) and atypical parkinsonian disorders (Hansson et al. 2017), making blood NfL a global marker of neurodegeneration rather than a disease-specific biomarker per se. Intriguingly, plasma NfL correlates highly with CSF NfL in the same individuals (correlation coefficients > 0.75 (Zetterberg and Blennow 2018)), further supporting the notion that plasma NfL indeed reflects central nervous system damage and could be used as a proxy measure for CSF NfL. However, at this time, no evidence has been gathered to suggest if blood NfL reflects the degree of neuropathology at *post-mortem*.

In this study, we sought to investigate this relationship by measuring longitudinal plasma NfL concentrations in cognitively unimpaired elderly controls (CTL) and patients with a clinical diagnosis of probable AD. For all participants, Braak staging of tau pathology was performed at *post-mortem*. Biochemical measures of brain Aβ (Aβ_1–42_, Aβ_1–40_ and Aβ_1–42_/ Aβ_1–40_) and tau (P-tau and T-tau), as well as immunohistochemical assessment of NfL, were performed in a subset.

## Materials and methods

### Study participants

The current study is comprised of two cohorts; the Alzheimer Research Trust (ART) cohort and the dementia case register (DCR). The details regarding the process of recruitment and clinical diagnosis have been previously described (Hye et al. 2006, 2014). In brief, the diagnosis of probable AD was made according to *Diagnostic and Statistical Manual for Mental Diagnosis*, fourth edition and National Institute of Neurological, Communicative Disorders and Stroke–Alzheimer’s disease and Related Disorders Association (NINCDS-ADRDA) clinical criteria. Standardized clinical assessments included the informant interview for diagnosis and the Mini-Mental State Examination (MMSE). CTL subjects were either spouses of cases or recruited though primary care age-sex registers; all had MMSE > 26. The *APOE* single nucleotide polymorphisms (SNPs) rs429358 and rs7412 were genotyped using Taqman SNP genotyping assays (determined by allelic discrimination assays based on fluorogenic 5′ nuclease activity) and the allele inferred. The human biological samples were sourced ethically, and their research use was in accordance with the terms of the informed consent. To be included in the present study, participants must have completed one or more blood draws throughout life and have reached *post-mortem* with a pathological assessment at the MRC London Neurodegenerative Diseases Brain Bank. We included 72 participants meeting such criteria (Table 1). Consent for autopsy, neuropathological assessment and research was obtained for all cases and the study was carried out under the ethical approval of the tissue bank. Block taking and neuropathological assessment was performed according to standard criteria (for more detail see (Skogseth et al. 2017)). This included assessment for vascular lesions, TAR DNA-binding protein 43 (TDP-43), and Lewy body pathology. Using these individual scores, a cumulative co-pathology burden score was generated for each subject (Additional file 1: Table S1). Furthermore, frozen brain tissue (20 mg, medial temporal gyrus [MTG]) was obtained for further biomarker characterization.

**Table 1:** Demographic, clinical (time point 1, 2 & 3) and *post-mortem* characteristics

		CTL			AD		
Time point		1	2	3	1	2	3
No.		15	12	4	57	57	32
Age, years		86 [79, 87]	88 [83, 90]	90 [89, 95]	83 [77, 88]	84 [78, 89]	87 [79, 89]
Gender, M/F		6/9	7/5	3/1	22/35	22/35	11/21
MMSE		29 [28.5, 30]	29 [28, 30]	29 [28, 29.3]	17 [9, 22]	14 [6, 20]	8 [4, 15]
*APOE*, ε4 carriers		5/14 (36%)	5/12 (42%)	2/4 (50%)	33/54 (61%)	33/54 (61%)	19/32 (59%)
NfL, pg/mL		29 [23, 34.5]	33 [29.3, 47]	39.3 [33, 45]	42 [33, 57]	48.3 [32, 67]	65 [47.3, 87]
Time to *post-mortem*, years		7 [3, 8]	7 [4, 8]	6.5 [6, 8]	4 [3, 6]	3 [2, 5]	2 [1, 3]
Braak staging	Transentorhinal (I/II)	8	7	3	2	2	0
	Limbic (III/IV)	4	3	0	11	11	4
	Isocortical (V/VI)	3	2	1	44	44	28

MMSE was found to be lower among CTL subjects, as compared to AD, across all three time points (*p* < 0.001)

Plasma NfL was higher among AD subjects, relative to controls, at time point 1 (*p* < 0.01) and at both second and third time points (*p* < 0.05)

### Plasma NfL

At the time of assessment, approximately 10 mL venous blood was collected in glass tubes containing sodium ethylenediaminetetraacetic acid (EDTA) from each subject. Participants were required to fast for at least 2 h prior to collection. The blood samples were centrifuged at 2000 x g at 4 °C for 8 min within approximately 2 h of collection. Plasma supernatant was collected, divided into aliquots, and frozen at − 80 °C until further use. Plasma NfL concentration was measured using the Simoa platform (NF-light; Quanterix, Lexington, MA) at the Maurice Wohl Clinical Neuroscience Institute, London, UK. Samples were randomized, blinded and measured in duplicate using a batch of reagents from the same lot. The intra-assay and inter-assay coefficients of variance were 8.1 and 11.2% respectively. The limit of detection (LOD) was 0.52 pg/mL and the lower limit of quantification (LLOQ) was 3.26 pg/mL when compensated for a 4-fold sample dilution.

### Brain tissue homogenization and biochemical measures (Aβ_1–42,_ Aβ_1–40,_ P-tau and T-tau)

Frozen *post-mortem* tissue (20 mg) from the MTG and subjacent white matter representing Brodmann area 22 (BA22) was obtained for 26 participants with serial plasma measures. Each sample was homogenized in 10% (*w*/*v*) Tris-buffered saline (TBS + protease inhibitors, cOmplete™, Roche) and sonicated for 1 min using a sonicating probe at 4 °C. The tissue lysate underwent ultra-centrifugation at 100,000 x g for 1 h (4 °C) and the supernatant was collected (TBS fraction [T]). Two % (*w*/*v*) sodium dodecyl sulfate (SDS) was added to the remaining pellet that was sonicated further for 1 min at 4 °C and centrifuged at 25,000 g for 1 h (4 °C). The supernatant was collected as the SDS fraction [S]. Lastly, 70% (w/v) formic acid was added to the remaining pellet, sonicated and spun under the same conditions as the SDS fraction. Subsequently, the supernatant was collected and neutralized at 1:20 with 1 M Tris for immunoassay analysis (formic acid fraction [F]). Tissue homogenates (T, S and F) were analysed for Aβ_1–42,_ Aβ_1–40,_ P-tau and T-tau by the human Luminex 4-plex xMAP assay (Millipore; HNABTMAG-68 K) at the Maurice Wohl Clinical Neuroscience Institute, London, UK. Briefly, both fractions T and S were diluted 1:2 and fraction F was undiluted (post pH neutralization at 1:20). Median fluorescent intensity (MFI) was measured using the Luminex 200 using Xponent 3.1 software. Both raw absorbance and MFI was exported to Sigma plot (ver13; Systat software) for estimation of protein concentration using a five-parameter logistic fit. The intra- and inter-assay coefficients of variance for Aβ_1–40_ (8.2% & 16%), Aβ_1–42_ (6.1% & 16%), P-tau (6.7% & 7%) and T-tau (9.1% & 14%) respectively. Each individual fraction and the sum of all fractions were used for further statistical analysis.

### NfL immunohistochemistry & microscopy

Seven μm thick formalin-fixed paraffin-embedded MTG sections were deparaffinised with xylene and rehydrated in a graded series of industrial methylated spirit (IMS; 100, 95, 70%). Endogenous peroxidase was quenched with 0.7% *v*/v hydrogen peroxide in methanol. Heat-induced epitope retrieval (HIER) was performed with citric acid-based antigen unmasking solution (Vector Laboratories). Sections were blocked with 10% *v*/v normal goat serum (Vector Laboratories, S-1000) in Tris-buffered saline (TBS) with 0.25% *v*/v Triton X-100 (TBS-Tx 0.25%). Mouse anti-NfL chain (clone DA2; 1:200 dilution; Thermo Fisher Scientific, MA1–2010) with 1% v/v blocking serum in TBS with 0.1% v/v Triton X-100 (TBS-Tx 0.1%) was incubated with sections overnight at 4 °C. Biotinylated goat anti-mouse IgG (1:200 dilution; Vector Laboratories, BA-9200) in TBS with 0.1% v/v Tween 20 (TBS-T 0.1%) was applied on the sections for 1 h at RT. Sections were visualised with VECTASTAIN Elite ABC HRP Kit (Vector Laboratories) and 3,3′-diaminobenzidine tetrahydrochloride (DAB) Peroxidase (HRP) Substrate Kit (Vector Laboratories). Sections were counterstained in Mayer’s haematoxylin. Sections were dehydrated in increasing concentrations of IMS (70, 95, 100, 100%) and cleared with xylene. Sections were coverslipped with DPX mountant medium. Sections were scanned using the VS120 Virtual Slide Microscope system (Olympus). The density of NfL immunostaining was measured by the percentage of positive DAB staining across the entire tissue section using Visiopharm image analysis program.

### Statistical analysis

Levels of plasma NfL between groups were compared using Wilcoxon signed rank test. Plasma NfL by Braak stage was examined using one-way ANOVA on ranks, with *post-hoc* Wilcoxon signed rank tests where appropriate. Categorical variables were compared pairwise using Fischer’s exact test. Spearman’s rank-order correlation was used to assess the association between plasma NfL and *post-mortem* Braak staging, as well as the relationship between plasma NfL levels and their rate of change. Linear mixed models, including random intercepts at the subject level, were used to assess longitudinal changes in NfL levels, with linear models used to assess the relationship between plasma NfL and MMSE and between NfL and MTG immunostaining. Given the association between NfL and age, all analyses were adjusted for age at *post-mortem* as well as for *post-mortem* delay, the interval between plasma sampling and death, and co-pathology burden. Four AD patients were excluded from our analyses due to outlying plasma NfL values (> 3 standard deviations of the mean). Analyses were performed using R (v.3.3.2, R Foundation for Statistical Computing, https://www.R-project.org/), with *p* < 0.05 (two-tailed) used to indicate statistical significance.

## Results

**Fig. 1:**
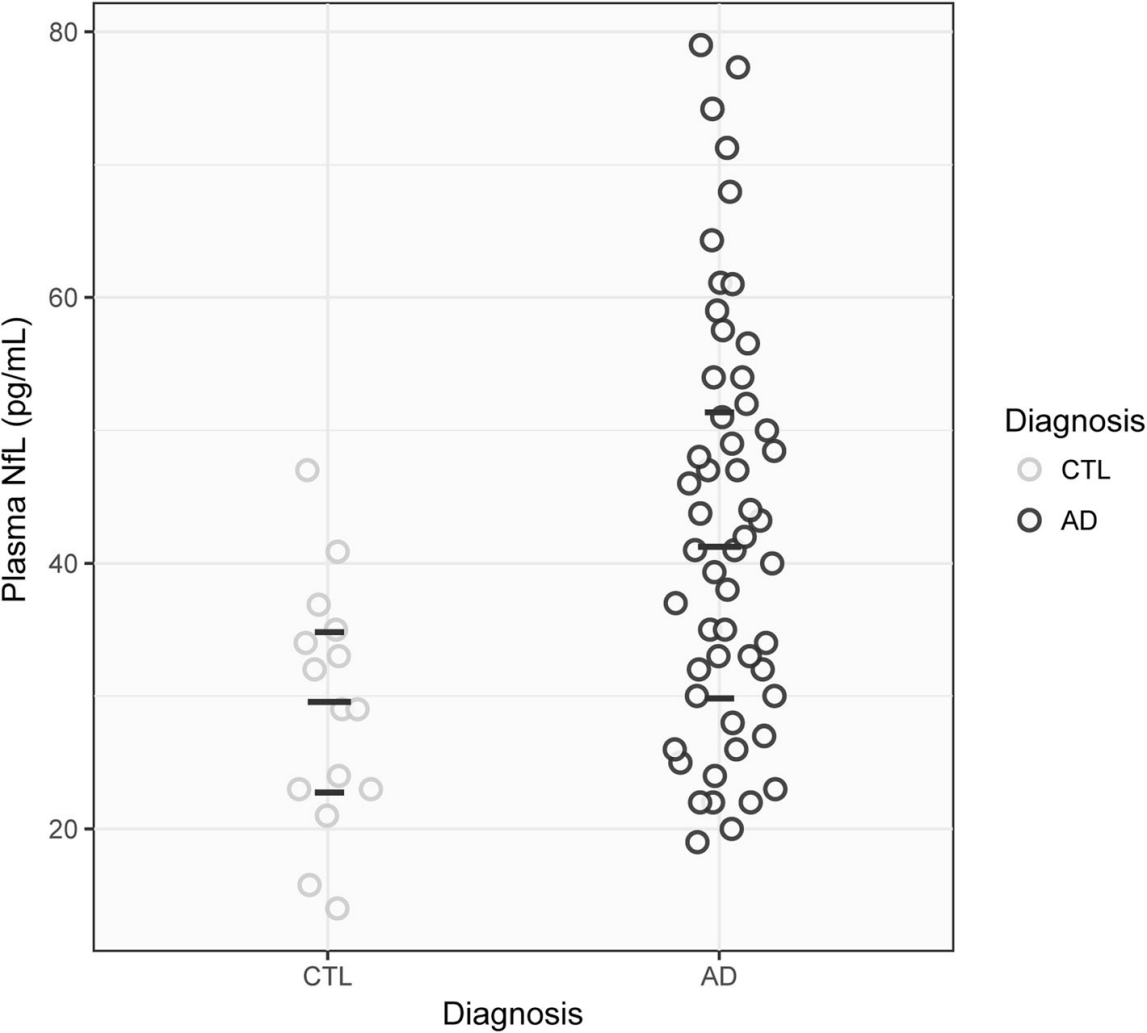
Plasma neurofilament light chain (NfL) concentrations in healthy controls (CTL) versus Alzheimer’s disease (AD) patients at time point 1 (*p* < 0.001). The horizontal dashes indicate median (long) and quartiles (short)

Demographic, clinical, and *post-mortem* Braak staging characteristics are summarized in Table 1. No significant between group differences were found for age at baseline, gender, *APOE* ε4 carriership, plasma storage time or in the interval to *post-mortem*. As expected, MMSE scores were lower in AD patients, as compared to CTL subjects, at both baseline and longitudinally (*p* < 0.001). Plasma NfL concentrations were significantly higher in AD, as compared to CTL subjects, at both time point 1 (*p* < 0.01, Fig. 1) and subsequent time points (*p* < 0.05).

### Plasma NfL correlates with age and with poorer cognition independent of age

**Fig. 2:**
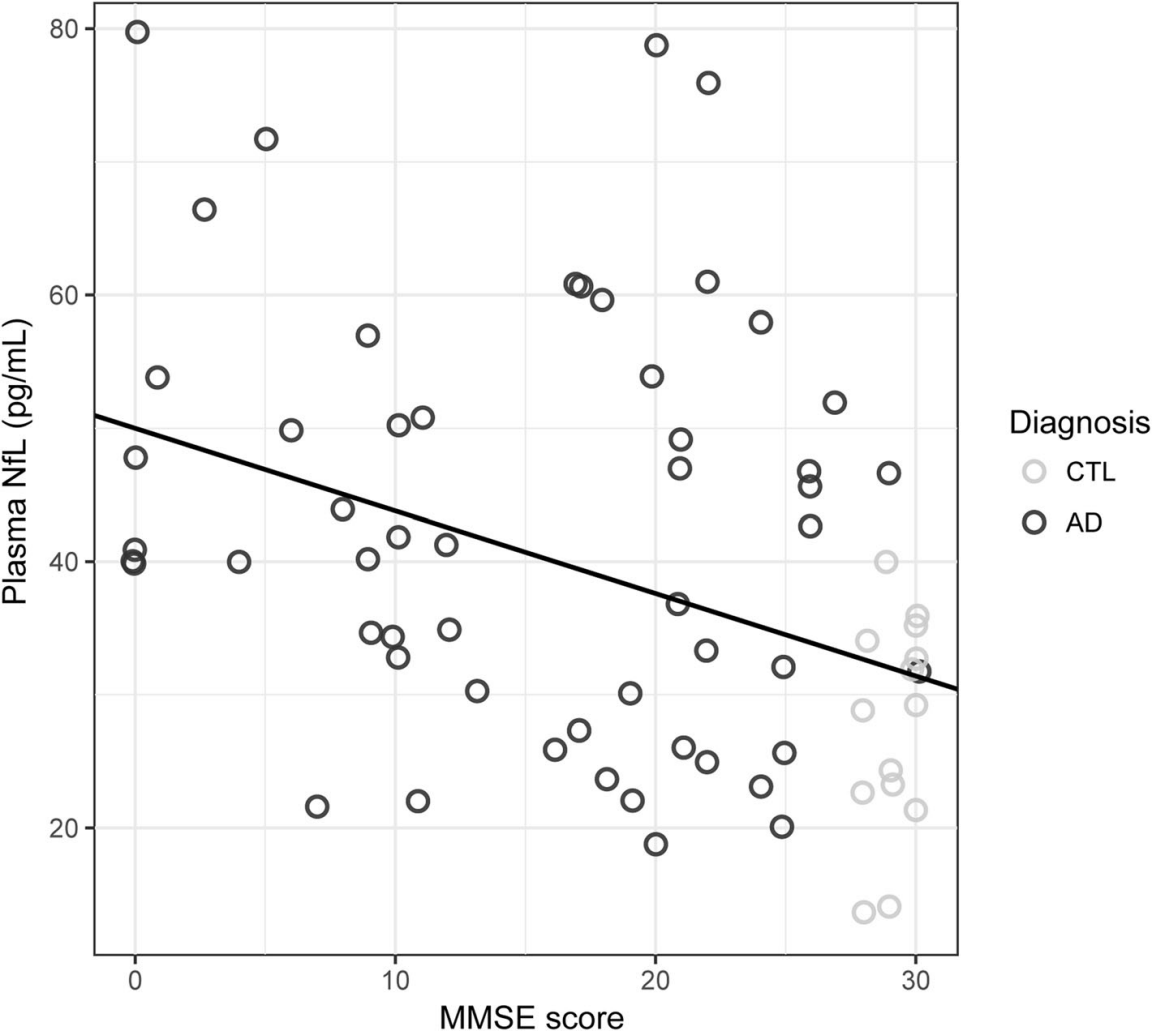
Time point 1 plasma neurofilament light chain (NfL) concentrations in comparison to global cognition (MMSE; rho = − 0.49, *p* < 0.001)

Across all subjects, plasma concentrations of NfL positively correlated with age (rho = 0.27, *p* < 0.05), although the correlation coefficient was stronger in CTL participants than AD participants. Due to this effect, a finding also reported in previous studies (Hansson et al. 2017; Zetterberg et al. 2016), further statistical analyses were adjusted for age. As expected, poorer MMSE scores were associated with increasing *post-mortem* Braak stages across all subjects (rho = − 0.61, *p* < 0.001); among sub-groups, statistical significance of this correlation held only among AD subjects (rho = − 0.30, *p* < 0.05; CTL, rho = − 0.33, *p* = 0.18). MMSE scores also showed a moderately strong inverse correlation with plasma NfL at time point 1 (rho = − 0.49, *p* < 0.001; Fig. 2). Time point 1 NfL was also associated with cognitive decline, as evidence by correlations with MMSE at time points 2 (rho = − 0.46, *p* < 0.001) and 3 (rho = − 0.31, *p* < 0.05). These associations, however, were not significant at the sub-group level.

### Plasma NfL is associated with *post-mortem* Braak staging

**Fig. 3:**
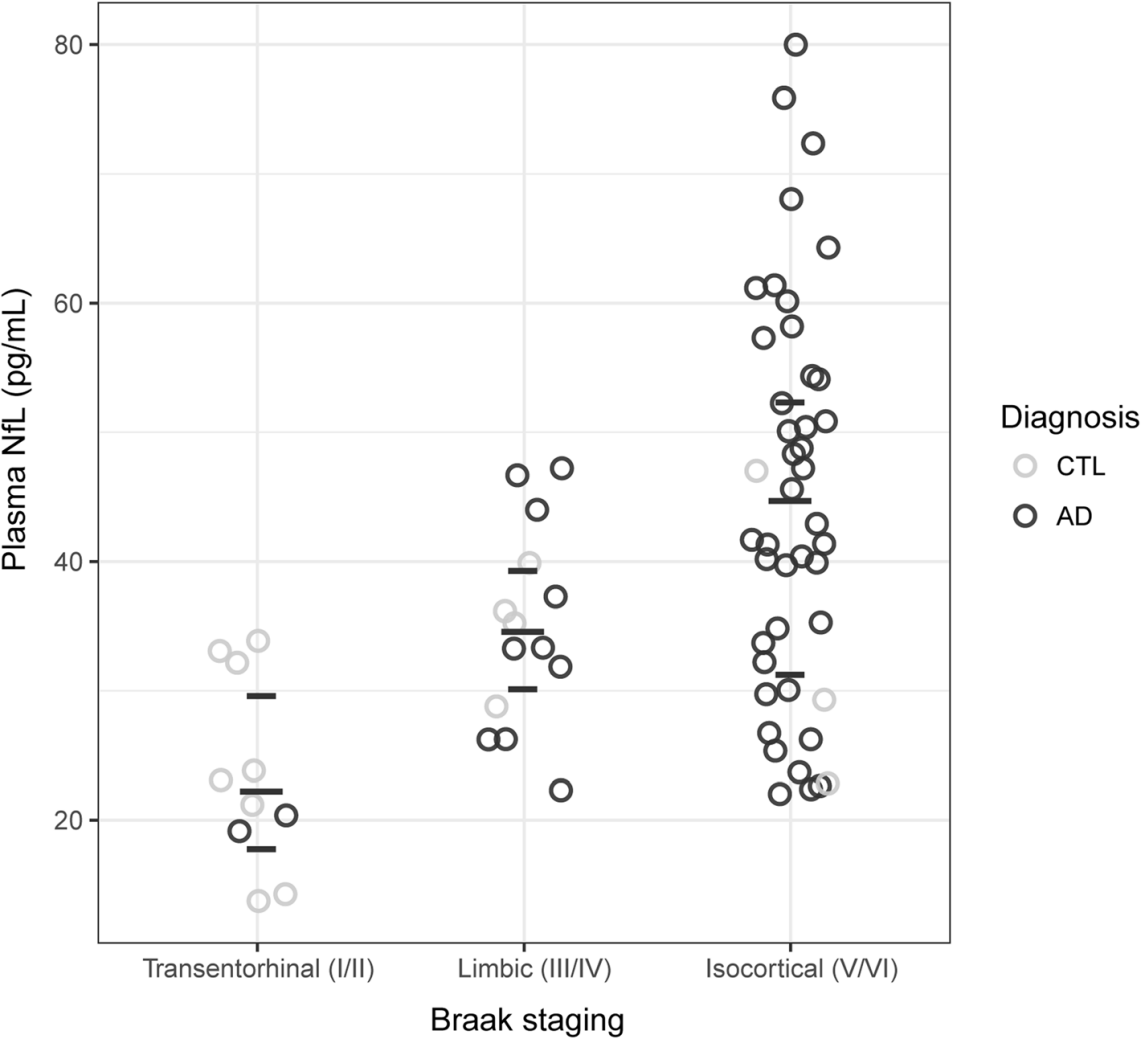
Time point 1 plasma neurofilament light chain (NfL) concentrations and Braak staging at post-mortem. The horizontal dashes indicate median (long) and quartiles (short). Clinical classification was determined at plasma time point 1

Using plasma NfL across all subjects at time point 1, a moderately strong correlation was observed with *post-mortem* Braak stages (rho = 0.55, *p* < 0.001); further, NfL levels were found to significantly increase across Braak stages (isocortical (*V*/VI) > limbic (III/IV) > transentorhinal (I/II); *p* < 0.001; Fig. 3). Longitudinally, patients with higher NfL concentration displayed higher Braak staging at *post-mortem* (*p* < 0.001).

### Longitudinal increases in plasma NfL

**Fig. 4:**
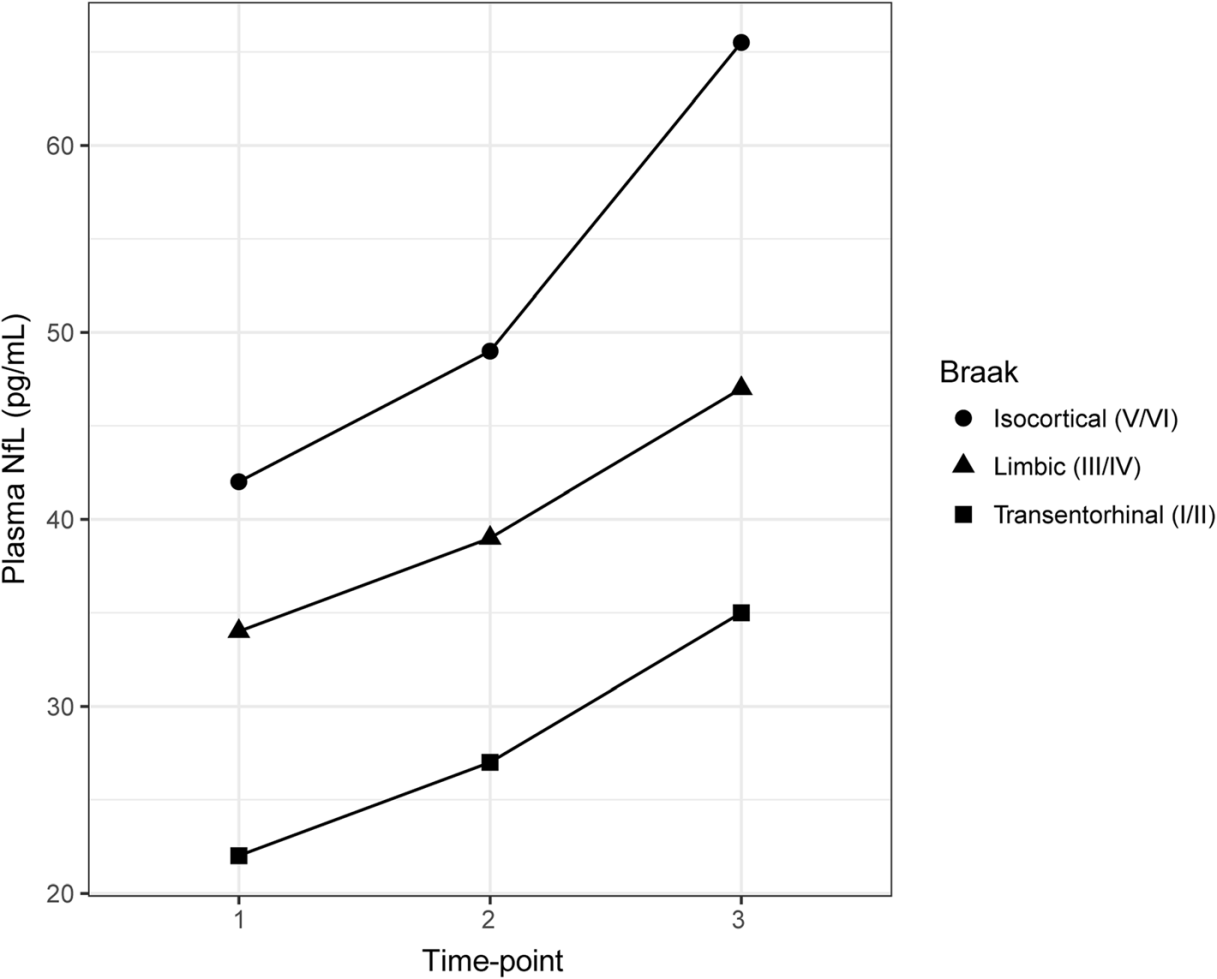
Longitudinal neurofilament light chain (NfL) concentrations by Braak stage. Circles, triangles, and squares indicate median values for isocortical (Braak *V*/VI), limbic (Braak III/IV) and transentorhinal (Braak I/II) stages, respectively. Significant increases were seen across all groups, between time point 1 and subsequent time points (time point 2, *p* < 0.01; time point 3, *p* < 0.001)

Figure 4 shows median NfL values across time points, plotted by Braak stage. Using linear mixed modelling, longitudinal increases in plasma NfL were seen across all groups (*p* < 0.01). A significant negative association was found, however, between plasma NfL at time point 1 and longitudinal measures (NfL rate of change; rho = − 0.46, *p* < 0.01) and annual percentage increase (rho = − 0.42, *p* < 0.05). No significant differences were seen between Braak stage group for either of these longitudinal metrics.

### Relationship between plasma NfL and biochemical measures of Aβ and tau

MTG homogenate measures of Aβ_1–40_, Aβ_1–42_, Aβ_1–42_/Aβ_1–40_, P-tau, and T-tau were available for 26 subjects: 7 controls and 19 AD. Firstly, the sum of the concentrations from fractions T, S and F were examined. Aβ isoforms, alone (Aβ_1–40_, Aβ_1–42_) and in ratio (Aβ_1–42_/Aβ_1–40_) were higher and lower, respectively, in AD, as compared to controls (*p* < 0.001; Additional file 2: Table S2). P-tau was higher in AD, though T-tau was higher in controls (*p* < 0.001). Significant group differences were also seen for separate homogenate fractions (T, S and F); similar to findings using the sum of fractions, Aβ and P-tau fractions were higher in AD, while T-tau fractions were higher in controls. Among plasma NfL measurements, only baseline levels significantly associated to the sum of T-tau fractions (rho = − 0.45, *p* < 0.05) and its F fraction (rho = − 0.42, *p* < 0.05).

### Inverse relationship between plasma NfL and immunohistochemical staining of NfL at *post-mortem*

**Fig. 5:**
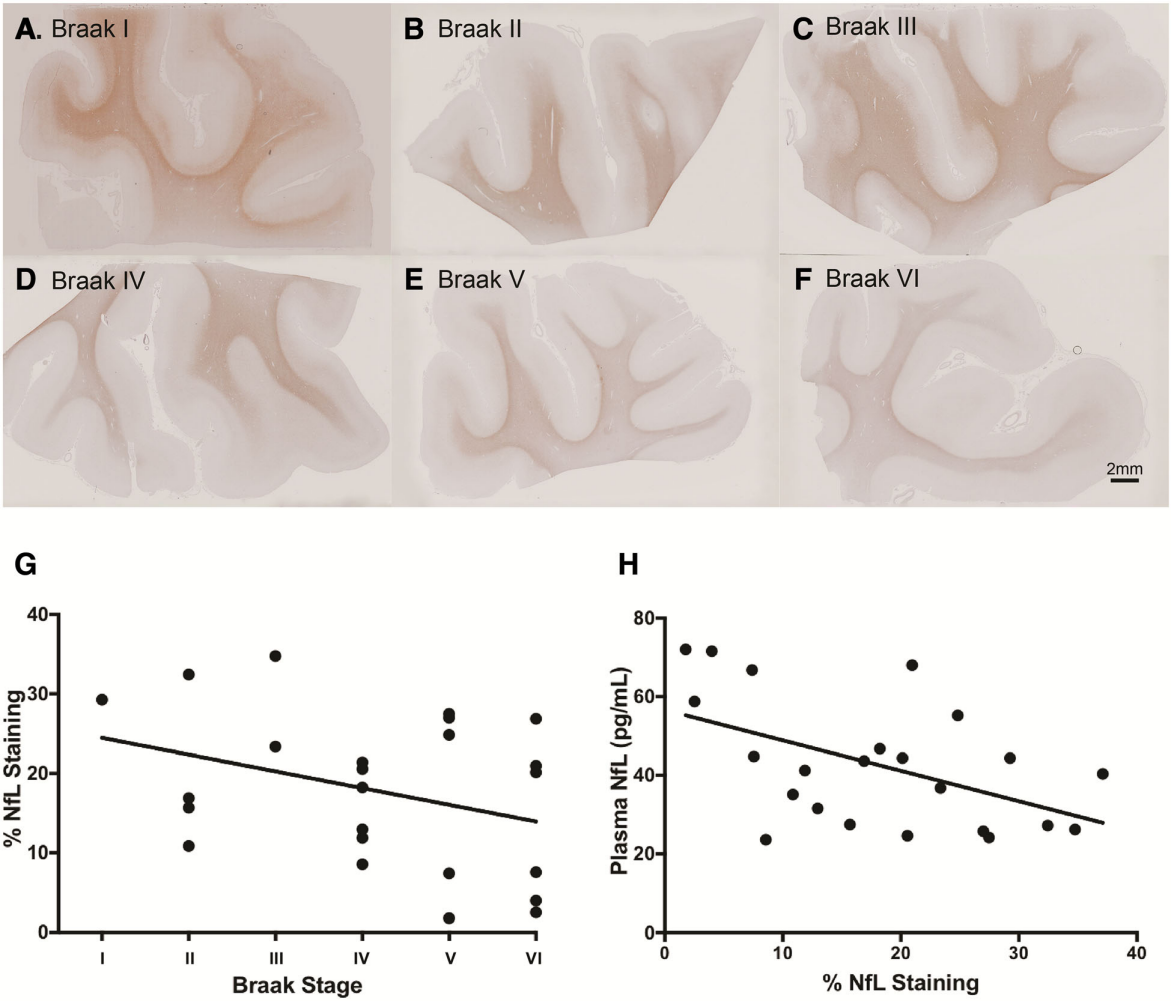
Formalin-fixed paraffin-embedded MTG sections stained for mouse anti-neurofilament light chain (clone DA2), one representative section from each Braak stage (I-VI) is displayed (**a**-**f**). The relationship between % NfL staining in the MTG and Braak staging at *post-mortem* (rho = − 0.34, *p* = 0.102) (**g**). A significant negative correlation between plasma NfL time point closest to death and % NfL staining in the MTG at *post-mortem* (rho = − 0.47, *p* < 0.01 (**h**)

Formalin-fixed paraffin-embedded MTG sections for 26 participants with longitudinal plasma NfL measures were immunohistochemically stained for NfL. Percentage of positive NfL staining (% staining) across the entire tissue section was measured using Visiopharm image analysis program. A non-significant trend of decreased % NfL staining with increased Braak staging was found (rho = − 0.34, *p* = 0.102; Fig. 5g). Visualised representation of decreasing % NfL staining from Braak stage I to VI are shown in Fig. 5a-f. Further, a significant association between increased plasma NfL and decreased % NfL staining was observed (rho = − 0.47, *p* < 0.01; Fig. 5h). This was only true for plasma NfL concentrations measured at time point 3, however (i.e., closest to *post-mortem*, median interval 2 years (interquartile range: 1, 3)). No association was found for plasma NfL at time point 1 or time point 2.

## Discussion

To our knowledge this is the first study investigating longitudinal measures of plasma NfL in relation to *post-mortem* measures, including neurofibrillary tangle (NFT) burden and neurodegeneration as determined by NfL immunohistochemistry. The main findings demonstrate that plasma NfL (1) is elevated with increased severity of AD-related NFT pathology at *post-mortem*, (2) increases over time independent of tangle pathology at *post-mortem* and (3) is inversely associated with the degree of NfL immunostaining at *post-mortem*. We also confirm other findings previously reported; plasma NfL correlates with age (Chatterjee et al. 2018; Gisslen et al. 2016; Lewczuk et al. 2018; Mattsson et al. 2017), however in our study this is driven by cognitively unimpaired individuals. Independent of age, plasma NfL correlates inversely with global cognition (Chatterjee et al. 2018; Lewczuk et al. 2018; Mattsson et al. 2017) and is increased in AD, despite large overlaps with other diagnostic groups (Lewczuk et al. 2018; Mattsson et al. 2017). Together, these findings support the notion that plasma NfL is a promising biomarker for neurodegeneration in AD.

There are 4 different neurofilament subunits, including NfL, neurofilament medium, neurofilament heavy and α-internexin. The neurofilaments are critical structural components of the neural cytoskeleton but their interaction with mitochondria, microtubules and other proteins (Yuan et al. 2017) imply their function is far more imperative than simply axonal stability. In addition to widespread grey matter loss, substantial white matter (WM) atrophy is also reported in AD (Delbeuck et al. 2003). These degenerated tracts are composed of large-caliber myelinated axons that are abundant in neurofilaments (Trojanowski et al. 1986). In previous studies, CSF measures of NfL have been shown to reflect WM changes (Sjogren et al. 2001; Zetterberg et al. 2016). Given the strong correlation of blood and CSF NfL (Disanto et al. 2017; Gaiottino et al. 2013; Mattsson et al. 2016) maybe it is unsurprising that we demonstrate blood NfL to reflect the state of degenerated large-caliber axons at *post-mortem*. However, this was only observed in plasma measures at the closest measure to death, and not at the first time point collection. This may suggest that this is a peripheral change in advanced dementia. Interestingly, time point 1 plasma NfL (approximately 8 years prior to death) was the highest in subjects classified as Braak *V*/VI at *post-mortem*, despite large group overlaps. We were also able to demonstrate that individuals classified as Braak III/IV had higher levels of plasma NfL than Braak I/II. This association remained at all subsequent time point plasma collections. Moreover, our only significant finding with biochemical brain analysis of core AD biomarkers was an association was between plasma NfL and P-tau. In support of these finding, plasma and CSF NfL concentrations have been shown to correlate with CSF P-tau, the key component of NFTs, in AD (Mattsson et al. 2017; Zetterberg et al. 2016) but not in parkinsonian disorders (Hansson et al. 2017). However, it remains unclear if CSF P-tau truly reflects NFT burden, with mixed findings having been reported for P-tau_181_*(*Buerger et al. 2007; Engelborghs et al. 2007; Seppala et al. 2012), though a positive association has been reported for P-tau_231_ (Buerger et al. 2006).

While cross-sectional plasma NfL has been shown to predict future cognitive decline and correlate with age, as we have also confirmed, there has been little evidence to determine how plasma NfL concentrations change over-time in AD. In this study, longitudinal analysis from 69 participants demonstrates increases of NfL concentration over time, independent of diagnosis and *post-mortem* Braak stage stratification. This finding is likely due to the on-going axonal injury that is associated with age-related changes. In CSF, NfL normal reference ranges increase 2.5-fold between 20 years and 50 years, doubling by the age of 70 years (Yilmaz et al. 2017). Furthermore, studies in healthy ageing indicate that CSF NfL remains a predictor of hippocampal atrophy that is independent of age and other AD biomarkers (Idland et al. 2017). In this current study, negative associations were found between time point 1 plasma NfL concentrations in both its rate of change and annual percentage increase. One might expect a slower trajectory of NfL after initial increases in groups with more severe pathology (in this study, likely to be AD) at *post-mortem*, as this may well reflect a deceleration in neurodegeneration. Longitudinal decreases in CSF tau have also been reported, possibly indicating a deceleration in neurodegeneration due to substantial neuronal loss (Sutphen et al. 2018). This hypothesis has also gained support from recent stable isotope labelling kinetics (SILK) studies that track the turnover of tau in the human CNS (Sato et al. 2018). However, in this study, a slowed trajectory of plasma NfL was observed in all groups (including Braak I/II and controls) and therefore the reported aged-related reduction in CSF turnover (Reiber 1994) may be a better explanation of this finding.

In the clinical work-up of suspected neurodegenerative disease, NfL will most likely be of limited value from a differential diagnostic perspective (with the differentiation of typical idiopathic Parkinson's disease from atypical parkinsonian disorders being one potential exception (Hansson et al. 2017)) but could instead be used to determine disease intensity, predict progression or rule out on-going neurodegeneration for cognitive complaints. Our data also suggests that plasma NfL could provide prognostic information as to the extent of NFT involvement in disease progression, which might prove important if immunotherapies targeting pathological tau in AD become available. However, the most promising application of plasma NfL is in evaluating treatment response in AD, as seen in multiple sclerosis studies (Novakova et al. 2017), as recent animal studies using β-secretase inhibitors have shown reduction in plasma NfL (Bacioglu et al. 2016).

This study is not without limitations. Although the primary aim was to associate plasma NfL with *post-mortem* pathology, conclusions about plasma NfL in isolation are based upon a smaller sample size than previous investigations. Further, given the nature of the study and cohort, cross-sectional, longitudinal and *post-mortem* participants had a bias toward AD participants compared to aged controls. AD patients were elderly, with a majority showing severe dementia, particularly at later time points. As such, our observations surrounding NfL are confined to a late portion of the AD continuum, one potentially also confounded by brain pathologies other than amyloid-β and tau (e.g., α-synuclein, TDP-43 and vascular lesions) (James et al. 2016), which have been shown to increase in prevalence with age (Schneider et al. 2007) and involve brain regions not examined in this study. Though we attempted to control for this by adjusting our analyses for a cumulative measure of reported co-pathology, this included only selected pathologies. In addition, while a useful measure of global cognition, the MMSE was designed to measure cognitive deficits in patients in the mild-to-moderate stages of dementia, and has been found to be deficient in detecting more severe cognitive decline, tending to show floor effects in patients with profound dementia (Herrmann et al. 2007; Vellas et al. 2005).

## Conclusion

This study, for the first time, demonstrates that plasma NfL is not only reflective of CSF biomarker changes but also of NFT pathology and neurodegeneration (as determined by NfL) at *post-mortem*. This important finding adds further, yet unique, support to the use of plasma NfL as a simple, accessible screening tool to rule out on-going neurodegeneration in primary care and for population enrichment and response monitoring in clinical trials.

## Additional files

Additional file 1: **Table S1.** Visual assessment (present/absent) of selected co-pathologies. (DOCX 16 kb)

Additional file 2: **Table S2.** Biochemical measures of Aβ_1-40_, Aβ_1-42_, P-tau and T-tau (pg/mL) in MTG brain homogenate fractions (TBS [T], SDS [S] and Formic acid [F]) for both CTL and AD subjects. The total fraction is the sum of all homogenate fractions. (DOCX 15 kb)
