# Supplementary material for: Increased plasma neurofilament light chain concentration correlates with severity of post-mortem neurofibrillary tangle pathology and neurodegeneration
Source: Acta Neuropathol Commun. 2019 Jan 9;7:5. doi: 10.1186/s40478-018-0649-3 (PMC6327431; doi:10.1186/s40478-018-0649-3)
Supplement: Supplementary file 1 — Table S1. Visual assessment (present/absent) of selected co-pathologies. (DOCX 16 kb) [file 40478_2018_649_MOESM1_ESM.docx]

**Supplementary Table 1.** Visual assessment (present/absent) of selected co-pathologies

|  |  | No. | Vascular | TDP-43 | Lewy body | Cumulative |
| --- | --- | --- | --- | --- | --- | --- |
| Clinical diagnosis | CTL | 15 | 2 (13%) | 1 (7%) | 3 (20%) | 6 (40%) |
|  | AD | 57 | 14 (25%) | 13 (23%) | 15 (26%) | 42 (74%) |
| Braak staging | Transentorhinal (I/II) | 10 | 3 (30%) | 1 (10%) | 5 (50%) | 9 (90%) |
|  | Limbic (III/IV) | 15 | 3 (20%) | 4 (27%) | 3 (20%) | 10 (67%) |
|  | Isocortical (V/VI) | 47 | 10 (21%) | 9 (19%) | 10 (21%) | 29 (62%) |

Data are presented as No or No. (%). Clinical diagnoses are those made at time point 1.

The Cumulative column refers to the sum of the pathology columns.
