# Supplementary material for: Increased plasma neurofilament light chain concentration correlates with severity of post-mortem neurofibrillary tangle pathology and neurodegeneration
Source: Acta Neuropathol Commun. 2019 Jan 9;7:5. doi: 10.1186/s40478-018-0649-3 (PMC6327431; doi:10.1186/s40478-018-0649-3)
Supplement: Supplementary file 2 — Table S2. Biochemical measures of Aβ1-40, Aβ1-42, P-tau and T-tau (pg/mL) in MTG brain homogenate fractions (TBS [T], SDS [S] and Formic acid [F]) for both CTL and AD subjects. The total fraction is the sum of all homogenate fractions. (DOCX 15 kb) [file 40478_2018_649_MOESM2_ESM.docx]

**Supplementary Table 2.** Biochemical measures of Aβ1-40, Aβ1-42, P-tau and T-tau (pg/mL) in MTG brain homogenate fractions (TBS [T], SDS [S] and Formic acid [F]) for both CTL and AD subjects. The total fraction is the sum of all homogenate fractions.

| Biochemical measure | | Controls | AD |
| --- | --- | --- | --- |
| Aβ_1-40_ | T | 446 [218.3, 669.3] | 446.6 [192, 1578] |
|  | S | 1073 [812.8, 6924] | 1877 [509, 6984] ^a^ |
|  | F | 337.9 [114, 1477s] | 1259 [205, 5869] ^b^ |
|  | Total | 5452 [1858, 15732] | 8044 [2738, 13318] ^b^ |
| Aβ_1-42_ | T | 1903 [515, 2721] | 4323 [2468, 4980] ^d^ |
|  | S | 4372 [2028, 6164] | 2998 [1138, 4393] ^d^ |
|  | F | 178 [35, 1255] | 758 [331, 1470] ^d^ |
|  | Total | 7200 [3320, 10558] | 8306 [4160, 11707] ^d^ |
| Aβ_1-42/_ Aβ_1-40_ | — | 8.92 [2.9, 52] | 10.96 [5.1, 29.72] ^d^ |
| P-tau | T | 47 [31, 67] | 64 [27, 173] ^d^ |
|  | S | 19 [13, 38] | 59 [27, 94.2] ^d^ |
|  | F | 4.2 [2.6, 5.8] | 40.2 [10.1, 159] ^b^ |
|  | Total | 88 [64, 126] | 277 [99.4, 364.1] ^d^ |
| T-tau | T | 12799 [12798, 13202] | 12742 [8255, 13210] ^e^ |
|  | S | 5171 [4406, 8320] | 2570 [845, 8890.5] ^e^ |
|  | F | 319 [226, 346] | 328 [242, 477] ^e^ |
|  | Total | 19509 [17655, 21641] | 16369 [9564, 19283] ^e^ |

^a^ Higher in AD, relative to controls, p < 0.05; ^b^ Higher in AD, relative to controls, p < 0.01;

^c^ Lower in AD, relative to controls, p < 0.001; ^d^ Higher in AD, relative to controls, p < 0.001;

^e^ Higher in controls, relative to AD, p < 0.001
